# Humans rather than climate the primary cause of Pleistocene megafaunal extinction in Australia

**DOI:** 10.1038/ncomms14142

**Published:** 2017-01-20

**Authors:** Sander van der Kaars, Gifford H. Miller, Chris S. M. Turney, Ellyn J. Cook, Dirk Nürnberg, Joachim Schönfeld, A. Peter Kershaw, Scott J. Lehman

**Affiliations:** 1 School of Earth, Atmosphere and Environment, Monash University, Clayton, Victoria 3800, Australia; 2 Cluster Earth and Climate, Faculty of Earth and Life Sciences, Vrije Universiteit, 1081 HV Amsterdam, The Netherlands; 3 INSTAAR and Geological Sciences, University of Colorado, Boulder, Colorado 80309-0450, USA; 4 Department of Environment and Agriculture, Curtin University, Perth, Western Australia 6102, Australia; 5 Climate Change Research Centre, School of Biological, Earth and Environmental Sciences, University of New South Wales, Sydney, New South Wales 2052, Australia; 6 Palaeontology, Geobiology and Earth Archives Research Centre, School of Biological, Earth and Environmental Sciences, University of New South Wales, Sydney, New South Wales 2052, Australia; 7 GEOMAR Helmholtz Centre for Ocean Research Kiel, D-24148 Kiel, Germany

## Abstract

Environmental histories that span the last full glacial cycle and are representative of regional change in Australia are scarce, hampering assessment of environmental change preceding and concurrent with human dispersal on the continent ca. 47,000 years ago. Here we present a continuous 150,000-year record offshore south-western Australia and identify the timing of two critical late Pleistocene events: wide-scale ecosystem change and regional megafaunal population collapse. We establish that substantial changes in vegetation and fire regime occurred ∼70,000 years ago under a climate much drier than today. We record high levels of the dung fungus *Sporormiella*, a proxy for herbivore biomass, from 150,000 to 45,000 years ago, then a marked decline indicating megafaunal population collapse, from 45,000 to 43,100 years ago, placing the extinctions within 4,000 years of human dispersal across Australia. These findings rule out climate change, and implicate humans, as the primary extinction cause.

## Abstract (web summary)

Megafaunal extinction in Australia has been attributed to both climate change and human causation. Here, van der Kaars *et al*. present a 150,000 year record offshore southwest Australia in which they refine the timing and nature of regional ecosystem changes and megafaunal population collapse.

The planet lost the majority of its largest mammals, birds and reptiles outside of Africa during the late Quaternary (the past 130,000 years). Identifying the drivers of the global extinctions of these megafauna (animals with average weights >44 kg) remains controversial^1^, with climate fluctuations^2^,^3^,^4^,^5^, human impact through hunting^6^,^7^,^8^ and/or habitat alteration^9^,^10^, and/or a synergistic combination of climate and humans^11^,^12^,^13^ the commonly cited causes. The major challenge has been precisely aligning individual records that contain unambiguous and regionally representative signals of these different factors to test extinction hypotheses robustly. Nowhere is the issue more acute than in Australia, where 85% of the continent's large mammal species went extinct sometime shortly after 50 kyr ago^9^,^10^,^14^,^15^, approximately the same time that humans established their first firm presence around the continent^14^,^16^. Dating both human colonisation and megafaunal extinction in Australia has been hampered by the occurrence of both events at or beyond the reliable limits of radiocarbon dating^15^,^17^,^18^,^19^.

A major challenge that has emerged is that most data relevant to the debate come from sites scattered across the continent that capture local events and may not be representative of regional changes.

An alternative approach to the analysis of individual (local) sites is to capitalize on the regional averaging of proxy signals offered by marine sediment sequences from the continental shelf surrounding Australia. Marine sedimentary sequences have distinct advantages over most terrestrial records, including generally continuously deposition and they can be precisely aligned and dated using the global marine isotope stratigraphy, the latter providing complementary age control to radiocarbon-based chronological frameworks. Here we present a continuous, stratigraphically well-constrained 150,000-year multi-proxy record from a sediment core recovered ∼100 km offshore south-western Australia, in which we identify the timing and sequence of events of the most widespread and sustained regional ecosystem changes over the past 150,000 years and precisely date regional megafaunal extinction. South-west Australia experiences a seasonal delivery of south-westerly air masses (winter-dominated)^20^,^21^,^22^ that supports large, dense forests with a diverse biota^23^. Importantly, the region has some of the earliest evidence for human arrival on the continent^19^,^24^ and is a candidate refugium for large mammals during past periods of aridity^25^, making it an ideal location to explore the response of megafauna to climate changes and human activity.

We utilize the number of spores from the dung fungus *Sporormiella* as our proxy for herbivore biomass in the catchment feeding the core site (a land area of ∼10,000 km^2^). We record high levels of *Sporormiella* from 150 to 45 kyr ago, indicating megafaunal populations were present under a range of climatic conditions from the warm, wet, interglacials to the hyper-aridity of the penultimate glacial during the period 150–130 kyr ago, and, critically, during the Marine Isotope Stage (MIS) 5–4 transition characterized by substantial changes in vegetation and fire regime ∼70 kyr ago. Importantly, however, *Sporormiella* underwent a marked and irreversible decline from 45 to 43.1 kyr ago, indicating megafaunal population collapse, with extinction apparently complete within 4,000 years of human occupation of the continent. Our results provide the first stratigraphically constrained sequence of events for Australian megafaunal extinctions, rule out extreme aridity and habitat change as causal mechanisms, leaving human agency, specifically imperceptible overkill^7^ as the most probable extinction cause.

## Results

### Recovery and analysis of the Cape Pasley marine core

**Figure 1:**
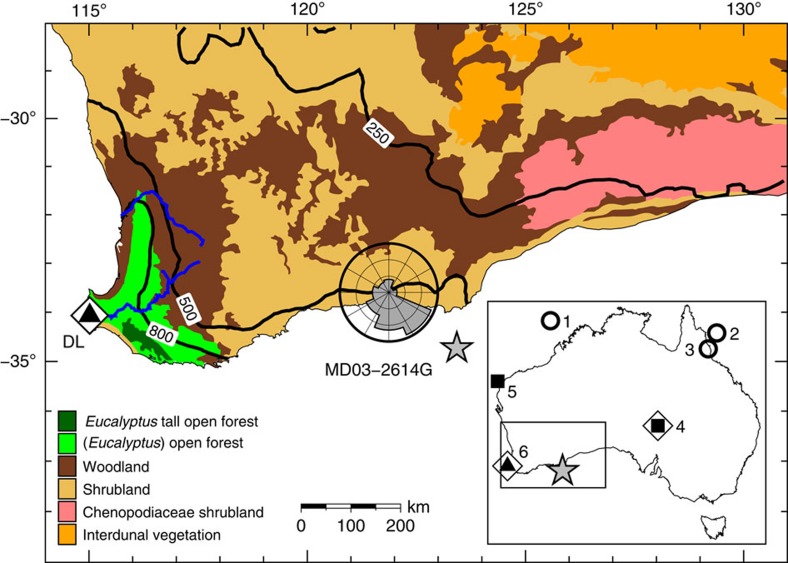
Map of south-western Australia showing location of core MD03-2614G (star) and archaeological and megafaunal site Devil's Lair (DL) discussed in text (triangle in open diamond). Shown in addition are main present-day vegetation types derived from AUSLIG^56^, 250, 500 and 800 mm annual rainfall isohyets derived from CRU CL 2.0 (ref. 57), wind direction rose for the town of Esperance obtained from the Bureau of Meteorology and main rivers (blue lines). The insert map of Australia shows all sites mentioned in the text: the three long (≥150 kyr) continuous records of environmental change are denoted by open circles, other records of environmental change by squares, megafaunal sites by open diamonds and the triangle denotes archaeological site. Site numbers on inset map: (1) North Australian Basin (core MD98-2167); (2) Coral Sea (core ODP Site 820); (3) Lynch's Crater; (4) Lake Eyre; (5) Cape Range Peninsula (core Fr10/95-GC17); (6) Devil's Lair. Maps made using GMT^58^.

**Figure 2:**
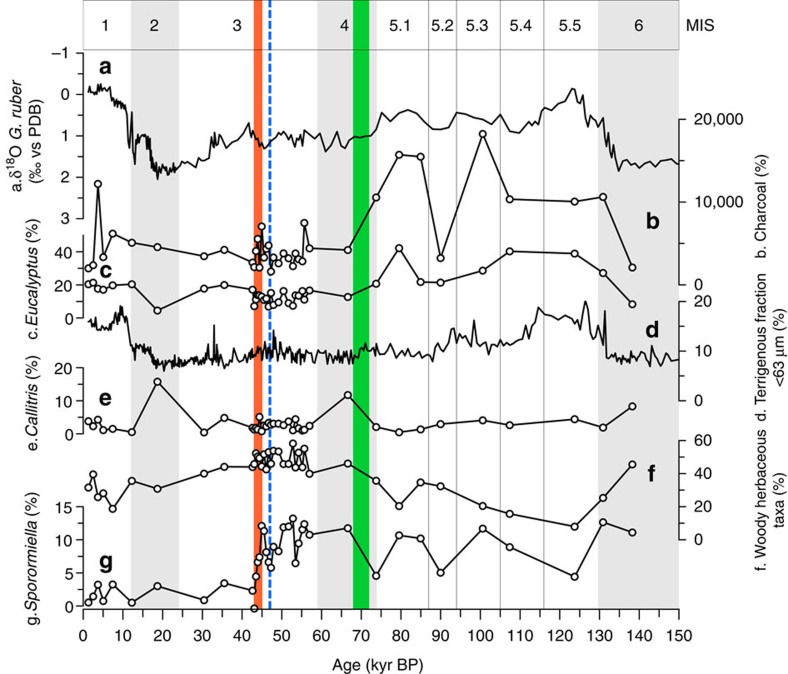
Multi-proxy diagram showing percentage values for selected pollen curves, charcoal and terrigenous sediment, as well as planktonic δ^18^O against age from core MD03-2614G. Marine isotope stages (MIS) are indicated. The timing of major environmental change in the record at ∼70 kyr ago is indicated by green shading, arrival of humans in Australia at ca. 47 kyr ago^16^,^19^ by blue dashed line and regional extinction of megafauna from 45 to 43.1 kyr ago in south-western Australia by red shading.

Gravity core MD03-2614G was recovered at 34° 43.728′ S and 123° 25.698′ E from a water depth of 1,070 m, ∼100 km south of Cape Pasley, south-western Australia, as part of the IMAGES program (Fig. 1). A well-constrained chronostratigraphy, established from tuning the planktonic δ^18^O record of *Globigerinoides ruber* to reference records and 11 accelerated mass spectrometry (AMS) ^14^C dates (Supplementary Tables 1 and 2; Supplementary Fig. 1) indicates that the 8.4 m core captures all of MIS 1–6, providing a continuous record of environmental change through the last ∼150 kyr (Fig 2 and Methods). In the absence of large river systems in the Cape Pasley area, delivery of terrigenous material to the site is by continental run-off and wind, the latter at present predominantly from the north to northwest (Fig. 1). Our environmental record was established through study of fossil pollen, charcoal, *Sporormiella* dung spores and terrestrial sediment (Methods). Palynological sampling was designed to include all MIS and sub-stages, with targeted higher resolution (0.7 kyr per sample) analysis across the likely period of overlap between human settlement and megafauna (55–42 kyr ago; ref. 14).

### Environmental change in south-western Australia

The Cape Pasley marine core record provides a high-resolution record of environmental change from the south-west of Australia. This marine record shows that the region experienced substantial environmental change during the past 150 kyr (Fig. 2; Supplementary Fig. 3). Overall, vegetation change has followed Northern Hemisphere ice-volume variation with expansion of tree cover under warm, high-precipitation regimes during MIS 5 and, to a lesser degree, MIS 1, and contraction during maximum cold and dry phases of MIS 6 2 (Fig. 2; Supplementary Fig. 3). The greatest density of forest, dominated by *Eucalyptus*, is identified during MIS 5.5 (the last interglacial), most likely developed under climatic conditions comparable to those today in the south-western corner of the continent (Fig. 1) and indicates that this period experienced the highest effective precipitation of the entire record. Marked and sustained decreases in percentages of woody elements, principally *Eucalyptus*, and increases in woody herbaceous scrub taxa, principally Asteraceae and Chenopodiaceae (Fig. 2; Supplementary Fig. 3), date from the MIS 5–4 transition at ∼70 kyr ago in response to regional drying. Generally, low terrestrial sediment input between 70 and 15 kyr ago also provides evidence for drier climatic conditions (Fig. 2). The records from the North Australian Basin and tropical Queensland (Lynch's Crater and ODP Site 820) record similar changes to drier vegetation types through this period^26^,^27^,^28^ and these environmental changes are broadly coincident with the hydrological changes inferred from the Lake Eyre sedimentary record^29^.

Before ∼70 kyr ago, charcoal peaks in the Cape Pasley record are associated with high values for Myrtaceous elements (Fig. 2; Supplementary Fig. 4), indicating that sclerophyll forests were the principal source of fuel for fires. After ∼70 kyr ago, lower and more stable values for charcoal indicate a reduction in biomass burning and that burning events were less extreme. This altered fire regime coincides with the change to more open vegetation shown by the expansion of herbaceous and herbaceous taxa and reduced terrestrial sediment levels (Fig. 2; Supplementary Figs 3 and 4). The record from the Cape Range Peninsula also shows high levels of charcoal associated with peak Myrtaceae values in the early part of the last glacial cycle (Fig. 1)^30^ before changing to a sparsely vegetated landscape with reduced biomass burning (Supplementary Fig. 4). Contrary to the previously reported environmental impacts recorded in connection with human arrival in the north of the continent^13^,^31^, the Cape Pasley record shows no sustained change in charcoal flux coincident with initial human arrival (at 50–45 kyr ago).

### The timing of megafaunal extinction in south-western Australia

Spores of the coprophilous fungi *Sporormiella*, commonly produced on the dung of herbivores^32^ and shown in the fossil record to be consistent and reliable markers of Pleistocene megafaunal biomass (declining during mass extinctions and increasing during the introduction of domesticated livestock in recent centuries^33^,^34^), were identified in this study (Supplementary Fig. 2) and used to indicate the presence of megafaunal populations. As a marine sequence, the Cape Pasley record is not subject to local taphonomic biases that may occur and require consideration at terrestrial settings such as local moisture levels^35^, near-by herbivore activity^36^ and position of the core site within the basin^37^. In our record, *Sporormiella* is abundant throughout MIS 6, 5, 4 and the early part of MIS 3, and rare in the later part of MIS 3, 2 and MIS 1. Vegetation, oxygen isotope and terrigenous sediment records show that MIS 6 was at least as dry and cool as MIS 2. High *Sporormiella* percentages in MIS 6, however, indicate that megafauna numbers were not significantly impacted by these extreme climatic conditions. Before 45 kyr ago the *Sporormiella* value is 9.7%, but declines sharply to 2% by 43.1 kyr ago (Supplementary Table 4; Supplementary Fig. 5). Declines of *Sporormiella* abundances to <2% of the pollen sum have repeatedly been shown to mark late Pleistocene megafaunal extinctions in North America^38^,^39^,^40^ and late Holocene extinctions in Madagascar^34^. We therefore interpret the marked decline of *Sporormiella* immediately after 45 kyr ago in our record as indicating the collapse of the Pleistocene megafaunal population in south-western Australia with extinction complete by 43.1 kyr ago. This extinction event post dates the substantial climatically driven regional environmental changes at ∼70 kyr ago by ∼27,000 years and precedes the cold and aridity of MIS 2 by 13,000 years.

## Discussion

The observed timing of the extinction event we record in south-western Australia (from 45 to 43.1 kyr ago) is in accord with megafaunal extinction in other regions. Across the Australian arid zone, the extinction of the giant bird *Genyornis newtoni*^10^ occurred 45.7±2.5 kyr ago with over 200 sites that contain burnt egg shell indicative of human consumption of its eggs^9^,^41^,^42^. At the Wet Cave sequence from Naracoorte (South Australia)^43^, extinction of megafauna occurred by 45.3 kyr ago and extinction of megafauna at Lynch's Crater (north-east Queensland) by ∼41 kyr ago (ref. 44). These age estimates accord with the age for continent-wide extinction of megafauna of 46.4 kyr ago (confidence interval 51–40 kyr ago) derived from OSL and U-series ages of articulated remains at 28 sites^15^. Importantly, the extinction event we identify in south-western Australia commences 2,000 years after the established age for human dispersal across Australia at ca. 47 kyr ago (ref. 16) and is mid-range within the estimate for megafauna–human overlap on mainland Australia centred on ∼44 kyr ago (ref. 14). The recent discovery of *Diprotodon* bone and eggshells of *Geyornis* in a well-stratified archaeological sequence at Warratyi rock shelter in central Australia dated to ≥49–46 kyr ago includes evidence for direct interaction between humans and megafauna in this time range^18^, supporting our results. Archaeological evidence from Devil's Lair (Fig. 1) shows human presence in south-west Australia by ∼48 kyr ago and regional human occupation by 45.5 kyr ago (ref. 19) with megafaunal extinction taking place at that site between 47 and 42 kyr ago (ref. 15).

**Figure 3:**
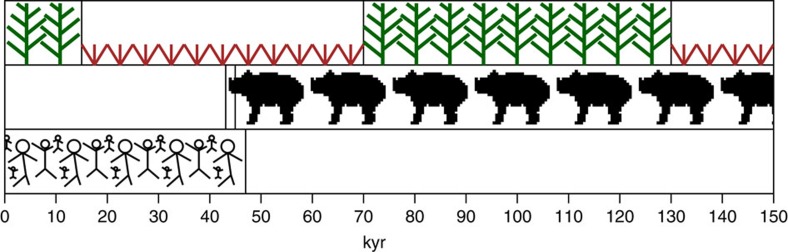
Schematic timeline derived from multi-proxy study of core MD03-2614G. Wide-scale ecosystem changes throughout the last glacial cycle (trees versus herbs) and regional megafaunal population collapse from 45 to 43.1 kyr ago (animal figures) in south-western Australia shown relative to time of human arrival on the Australian continent (stick people)^16^,^19^.

Our results provide the most refined stratigraphically constrained sequence of events for Australian megafaunal extinctions and the first regionally representative record of their loss. Our new data establish the precise timing and order of the most substantial, widespread and sustained environmental changes of the last glacial cycle, as well as megafaunal extinctions, in south-western Australia (Fig. 3). Specifically, they show that: (1) dense eucalypt woodland gave way to open Chenopodiaceae–Asteraceae shrubland at the MIS 5–4 transition at ∼70 kyr ago, a change initiated some ∼23,000 years before evidence for the presence of people on the continent, indicating that anthropogenic mechanisms were not responsible for the sustained change in vegetation; (2) there were few widespread fire events after 70 kyr ago, likely owing to the less flammable nature of the open vegetation; and (3) the regional collapse of megafaunal populations commenced at 45 kyr ago and took <2,000 years in total (concluded by 43.1 kyr ago).

We therefore conclude there is no temporal association between climatically driven environmental changes and megafaunal extinction in the south-west region. There is also no indication in our data that the megafauna suffered a slow demise commensurate with increases in aridity. Furthermore, our data show no impact on megafauna presence of the marked stepwise increases in aridity over the longer term (during a full glacial cycle). This work adds to a growing body of evidence showing similar timing for extinction in the south-west of Australia as for extinction at sites in central^9^,^10^, southern^36^ and north-eastern Australia^35^, as well as continent-wide extinction of all megafauna by ∼46 kyr ago (ref. 15).

The debate over megafaunal extinction causes has consistently been between climate and humans. Having eliminated climate as the primary cause of extinction, we turn to consideration of human causation, of which hunting is most favoured as extinction driver. In Australia, there are no reported kill sites to indicate concentrated hunting efforts (other than transient sites where humans gathered and consumed *Genyornis* eggs) or widespread slaughter of megafauna. However, this does not disprove that human hunting took place and is itself informative for shaping debate. Large mammals are demographically vulnerabile to hunting pressure owing to their typically low reproduction rates and low population growth^7^,^8^. It has been estimated that low intensity hunting (such as the killing of just one juvenile per person per decade) could result in a species being extinguished over a few hundred years^7^. Such selective, low-level hunting would be virtually imperceptible in the archaeological record, accounting for the lack of reported kill sites. In this scenario, all species of megafauna could have been lost from the entire continent within a few thousand years. In the Cape Pasley record, megafaunal population collapse commenced within 2,000 years of the established date for human dispersal on Australia at ∼47 kyr ago (ref. 16), with extinction completed within 4,000 years. We conclude our results are consistent with extinction being driven by such ‘imperceptible overkill'^7^.

## Methods

### Oxygen-isotope analysis

Sediment samples were dried at 50 °C, weighed and washed through a 63 μm mesh, dried and weighed again. The size fraction >250 μm was separated by dry sieving. On average, 10–12 specimens of the planktonic foraminifera species *G. ruber* (white) were picked from the >250 μm size fraction. Only clean foraminifera specimens of approximately the same size were selected. Measurements of stable oxygen and carbon isotopes were performed on a Thermo DeltaPlus mass spectrometer equipped with a GasBench 2 carbonate preparation device. The isotope values were calibrated versus NBS 19 (National Bureau of Standards) and the in-house standard ‘Standard Bremen' (Solnhofen limestone). Isotope values are reported in per mil (‰) relative to the VPDB (Vienna Pee Dee Belemnite) scale. The analytical precision (1−sigma value) as obtained from 11 replicate Standard Bremen measurements was on average 0.058‰ for δ^18^O and 0.044‰ for δ^18^C. Only the oxygen-isotope data are reported in this paper.

### Radiocarbon analysis

Eleven AMS ^14^C dates were obtained for core MD03-2614G principally within the period of *Sporormiella* decline (Supplementary Table 1). Sample KIA 22661 was comprised of Pterpoda taken from the >250 μm size fraction and was prepared at the Leibniz Laboratory for Radiometric Dating and Stable Isotope Research. The sample was treated with 15% H_2_O_2_ in an ultrasonic bath followed by 100% H_3_PO_4_ at 90 °C produce CO_2_. The CO_2_ was reduced with H_2_ at 600 °C using a Fe catalyst and the resulting ion–graphite mixture was packed into a target and measured. The following 10 samples were comprised of monospecific planktic foraminifera picked from the >250 μm size fraction of washed sediment. These 10 samples were prepared at the INSTAAR Laboratory for AMS Radiocarbon Preparation and Research (NSRL) before measurement by Accelerator Mass Spectrometry at the Keck Carbon Cycle AMS Laboratory at the UC Irvine (KCCAMS). Foraminifera were leached for 5 min in a 0.001 M solution of HCl. Each sample was then reacted with H_3_PO_4_ and the CO_2_ produced was cryogenically purified. The purified CO_2_ was reduced with H_2_ in the presence of a Fe catalyst and the resulting graphite was packed into AMS targets and measured.

### Chronology

*Tuning and tie-points*. The age model for core MD03-2614G was established by tuning the *G. ruber* δ^18^O curve from MD03-2614G to nearby planktonic δ^18^O records from the Great Australian Bight^45^,^46^, Vostock ice core Deuterium (ref. 47) and the globally stacked benthic δ^18^O record^48^ (Supplementary Table 2). Reference curves were chosen for core intervals of comparable resolution^49^. The *Globigerina bulloides* δ^18^O curve was well in phase from the last glacial maximum (LGM) to 37 kyr ago but offset below. Radiocarbon dates were given preference to accomplish the graphic correlation with different reference curves.

*Bayesian age modelling*. The planktonic δ^18^O and radiocarbon ages were used to develop an age model using a P_sequence deposition model in OxCal 4.2 (refs 50, 51) with General Outlier analysis detection (probability=0.05)^52^. The ^14^C ages were calibrated using the Marine13 calibration data set^17^. To accommodate possible changes in the marine reservoir age, a Delta_R of 91±54 ^14^C years was used (determined from the ^14^CHRONO Marine Reservoir Database at http://calib.org/marine/). Using Bayes' theorem, the algorithms employed sample possible solutions with a probability that is the product of the prior and likelihood probabilities. Taking into account the deposition model and the actual age measurements, the posterior probability densities quantify the most likely age distributions; the outlier option was used to detect ages that fall outside the calibration model for each group, and if necessary, down-weight their contribution to the final age estimates. Modelled ages are reported here as thousands of calendar years BP, kyr or ka and plotted against depth (Supplementary Fig. 1).

### Grain-size analysis

The terrigenous fraction of the sediment represents continental run-off and wind-blown dust in the <63 μm size fraction. The sand-sized fraction >63 μm is almost entirely composed of pelagic carbonate particles, such as planktonic foraminifera, pteropods and coccoliths, as well as benthic foraminifera and bryozoan debris. Bulk sediment samples were dried at 50 °C and finely ground in an agate mortar. Total organic and total carbon were analysed with a Leco CS200 furnace. The accuracy is 0.2% according to 52 parallel measurements of a commercial Leco standard. The proportion of organic carbon was subtracted from total carbon to calculate the carbonate content of the bulk sediment. The non-carbonate fraction was calculated as 100% CaCO_3_.

### Pollen preparation and counting

A total of 39 samples were analysed in this study. Samples of ∼4–5 cm^3^ each were processed using the following method. Sub-sampled sediment was suspended in ∼40 ml of 10% sodium pyrophosphate followed by sieving over 210 and 7 μm mesh. The sediment fraction retained was treated with 10% HCl then acetolysis (10 min) and heavy liquid separation (sodium polytungstate S.G. 2.0, 20 min at 2,000 r.p.m.; 2 × ). A known amount of *Lycopodium* spores was added to the samples before processing to allow calculation of pollen and charcoal concentrations. Slides were mounted with glycerol and sealed with paraffin wax. Pollen was generally well preserved and abundant throughout the core. Pollen was counted along evenly spaced transects at magnification of × 630 on a Zeiss Axioskop. The average number of pollen counted per sample was ∼230 grains. Pollen percentages were calculated on a pollen sum consisting of all angiosperms and gymnosperms pollen grains counted (that is, excluding pteridophyte and fungal spores).

### Charcoal analysis

Charcoal fragments larger than 10 μm were counted along three equally spaced transects of each sample slide and results presented as percentages of the dryland pollen sum.

### *Sporormiella* identification and counting

Identification of *Sporormiella* was made according to the following criteria. They are ascospores with a sigmoid germinal aperture extending the entire length of the cell. Cells are 12–28 μm × 9–15 μm and are present as two types; end cells show one flattened and one round end, middle cells show two flattened ends^53^ (Supplementary Fig. 2). *Sporormiella* spores were calculated as a percentage of the pollen sum.

### Palynological results and statistical analyses

A summary pollen diagram with the results of the palynological analysis is presented in Supplementary Fig. 3. Zonation of the pollen diagram was made using the outcome of stratigraphically constrained cluster analysis (CONISS routine)^54^. The 12 major pollen taxa (shown on Supplementary Fig. 3) were used in CONISS; all minor taxa, charcoal and *Sporormiella* were excluded. The main divisions of the diagram were placed at ∼70 and 15 kyr ago and the resulting zonation concurs with the per-eye interpretation of the pollen data and confirms that significant vegetation change took place at ∼70 kyr ago, coincident with the reduction in fire. The CONISS results also indicate that *Sporormiella* decline is not associated with major changes in vegetation.

Ramp function regression and bootstrap re-sampling (RAMPFIT)^55^ were applied to the *Sporormiella* data to quantify uncertainties. The RAMPFIT output confirms the per-eye interpretation of the *Sporormiella* curve and places the onset of the decline at 44.966±0.851 kyr ago and the completion at 43.087±0.645 kyr ago, with the number of *Sporormiella* spores decreasing from 9.694±0.412% to 2.041±0.729% (Supplementary Table 3, 4; Supplementary Fig. 5).

### Data availability

Data on the physical properties of core MD03-2614G are archived in the PANGAEA database (doi:10.1594/PANGAEA.131767). Radiocarbon dating was supported by the NSF's Archaeology and Archaeometry Program (BCS-0914821). All relevant data are available from the authors.

## Additional information

**How to cite this article:** van der Kaars, S. *et al*. Humans rather than climate the primary cause of Pleistocene megafaunal extinction in Australia. *Nat. Commun.* **8,** 14142 doi: 10.1038/ncomms14142 (2017).

**Publisher's note**: Springer Nature remains neutral with regard to jurisdictional claims in published maps and institutional affiliations.

## Supplementary Material

Supplementary Information Supplementary Figures 1-5 and Supplementary Tables 1-4
