# Supplementary material for: Humans rather than climate the primary cause of Pleistocene megafaunal extinction in Australia
Source: Nat Commun. 2017 Jan 20;8:14142. doi: 10.1038/ncomms14142 (PMC5263868; doi:10.1038/ncomms14142)
Supplement: Supplementary Information — Supplementary Figures 1-5 and Supplementary Tables 1-4 [file ncomms14142-s1.pdf]

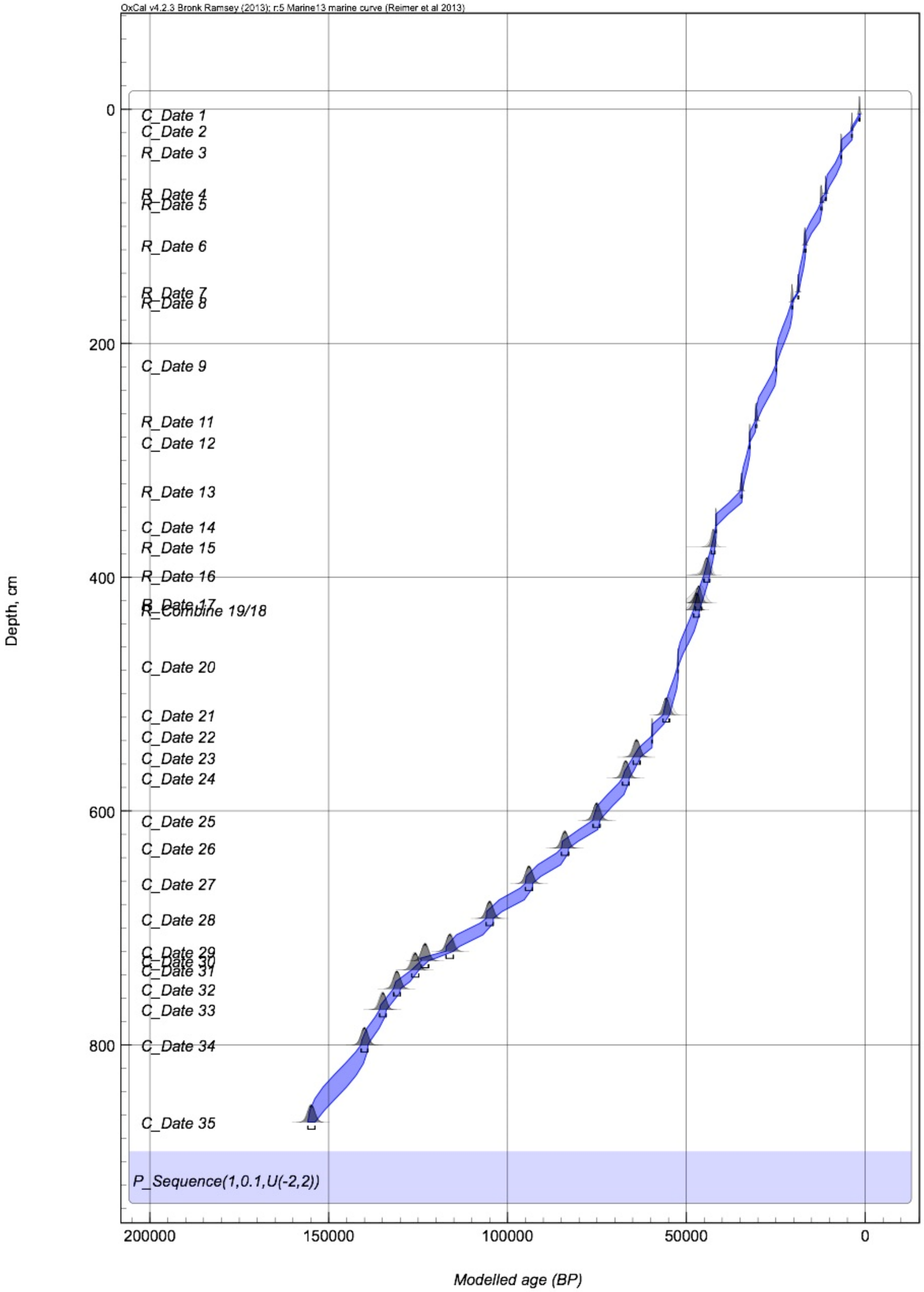

Supplementary Fig. 1. Modelled age-depth relationship in the Cape Pasley sequence using OxCal 4.1[51]. Blue envelope denotes the 1σ (68.2%) confidence limits. Light and dark grey curves denote the prior and posterior probability distributions, respectively.

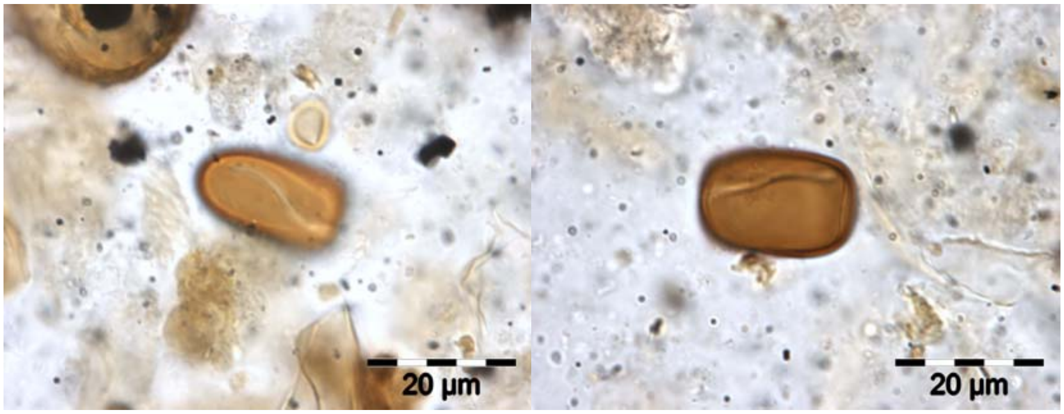

Supplementary Fig 2. Photographs of *Sporormiella* spores from core MD03-2614G depth 524 cm (~57 ka BP).

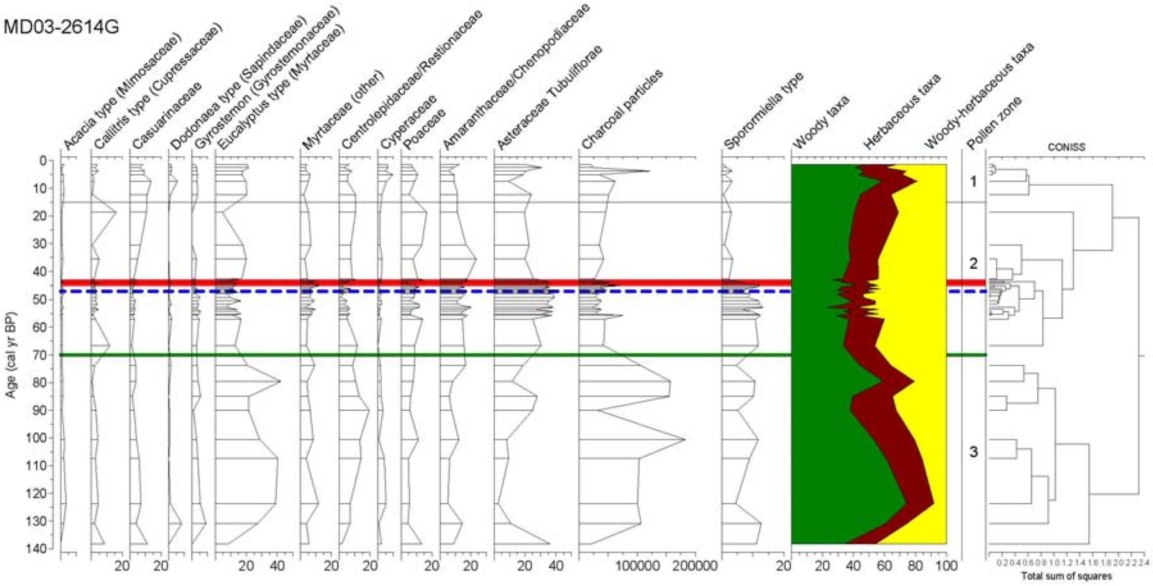

Supplementary Fig 3. Pollen diagram from core MD03-2614G showing 12 major pollen types (percentages of pollen sum), charcoal concentrations, *Sporormiella* spores (percentages of the pollen sum) and structural vegetation taxon group ratios. Zonation of the diagram is based on stratigraphically constrained cluster analysis (CONISS routine)[54] using the 12 major pollen types. The green line indicates major environmental change at ~70 ka, blue line the dispersal of humans on Australia *ca*. 47 ka[16,19] and red shading the regional extinction of megafauna between 45 and 43.1 ka in south-western Australia as indicated by the *Sporormiella* curve.

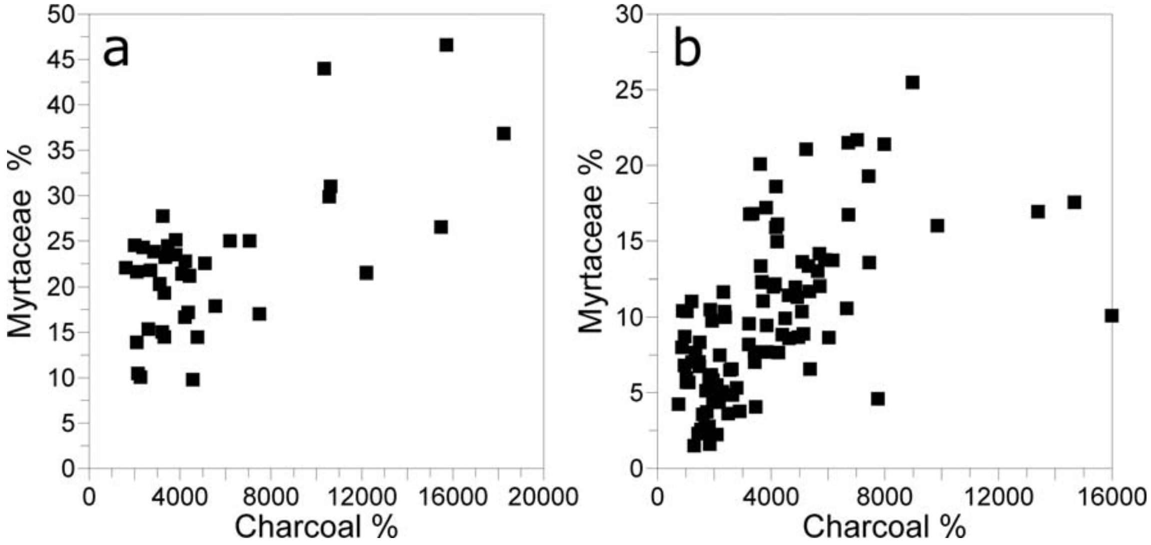

Supplementary Fig 4. Scatter plot showing percentage of charcoal in relation to percentage of Myrtaceae in core MD03-2614G (a) and Fr10/95-GC17 (b)[30].

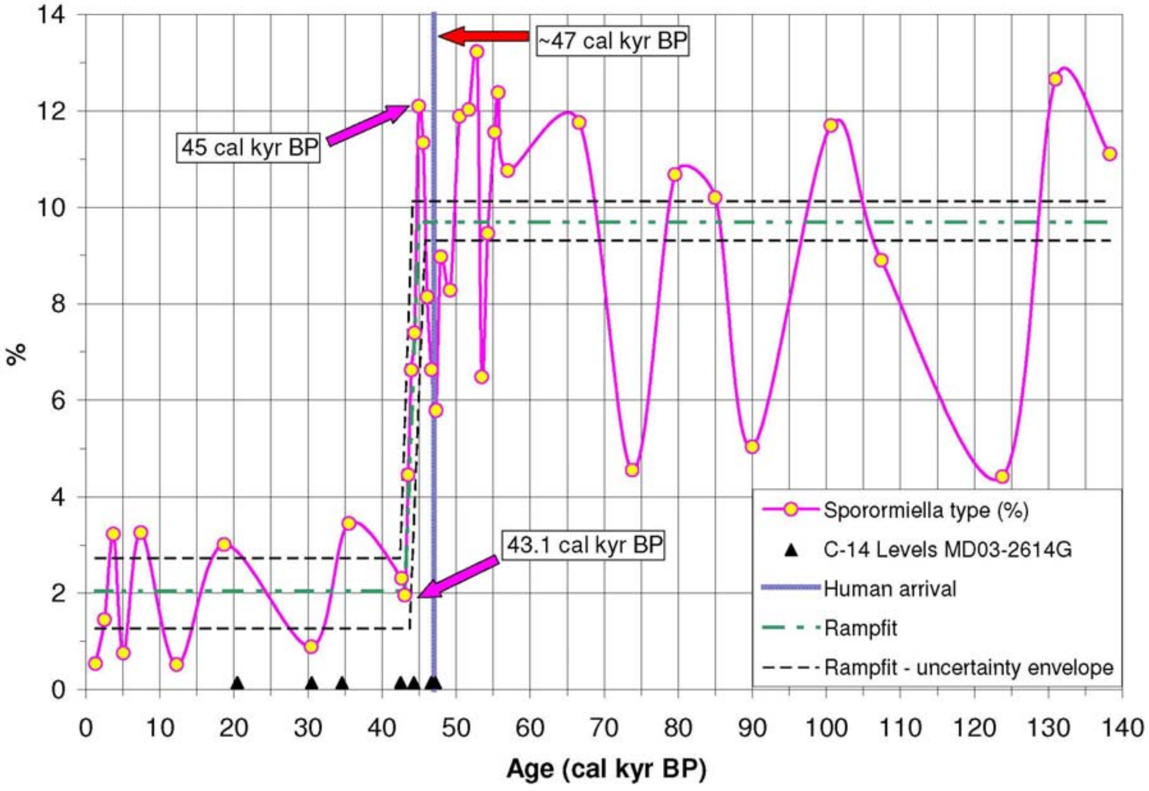

Supplementary Fig 5. Percentage-age plot for representation of *Sporormiella* spores from core MD03-2614G showing the results from the RAMPFIT routine[55] relative to established date of human arrival[16,19].

Supplementary Table 1. AMS ^{14}C results for core MD03-2614G listing sample levels, material analysed and laboratory codes.

level (cm)	Taxon	mass (mg)	size fraction	^{14}C LAB ID	^{14}C Age	± (1-sigma)
164.5	Pteropods	1.200	>250 µm	KIA 22661	17490	110
266	*G. bulloides*	7.580	>250 µm	CURL-16658	27500	170
266	*G. ruber*	6.786	>250 µm	CURL-16684	26650	150
326	*G. bulloides*	7.575	>250 µm	CURL-16677	31110	250
374	*G. bulloides*	7.264	>250 µm	CURL-16076	38950	960
398	*G. bulloides*	7.130	>250 µm	CURL-16069	40920	1230
398	*G. ruber*	7.004	>250 µm	CURL-16070	42950	1580
422	*G. bulloides*	6.790	>250 µm	CURL-16080	43350	1650
422	*G. ruber*	4.825	>250 µm	CURL-16072	38570	920
428	*G. bulloides*	11.300	>250 µm	CURL-16685	44630	1320
428	*G. ruber*	11.268	>250 µm	CURL-16661	44460	1280

Supplementary Table 2. Radiocarbon dates and tuning tie-points used to establish the chronology for core MD03-2614G.

Depth (cm)	Age (cal. ka)	Event, reference curve	Depth therein (m)
4	1.61	Correlation with[45], $\delta^{18}O$ *G. ruber* ODP 1127, GAB	0.3
18	3.73	Correlation with[45], $\delta^{18}O$ *G. ruber*. ruber ODP 1127, GAB	1
36	6.76	Correlation with[45] , $\delta^{18}O$ *G. ruber* ODP 1127, ^{14}C-dated	2
72	11.08	Correlation with[45] , $\delta^{18}O$ *G. ruber* ODP 1127, ^{14}C-dated	7.9
80	12.33	Correlation with[45], $\delta^{18}O$ *G. ruber* ODP 1127, ^{14}C-dated	8.41
116	16.28	Correlation with[45], $\delta^{18}O$ *G. ruber* ODP 1127, GAB; close to ^{14}C dating at 9.4 m: 16.63 cal. ka	9.32
156	18.84	Correlation with[46], $\delta^{18}O$ *G. bulloides*, MD03-2611; close to ^{14}C dating at 500 cm: 18.62-18.85 cal. ka)	5
164.5	20.72	Radiocarbon dating (pteropods, KIA22661, calibrated Marine13)	
218	24.86	Correlation with[47], δD, Vostok ice core	450
266	31.51	Radiocarbon dating (*G. bulloides*, CURL-16658, calibrated Marine13)	
266	31.13	Radiocarbon dating (*G. ruber*, CURL-16684, calibrated Marine13)	
284	32.30	Correlation with[47], δD, Vostok ice core	538
326	35.67	Radiocarbon dating (*G. bulloides*, CURL-16677, calibrated Marine13)	
356	41.71	Correlation with[47], δD, Vostok ice core	656
374	43.25	Radiocarbon dating (*G. bulloides*, CURL-16076, calibrated Marine13)	
398	44.62	Radiocarbon dating (*G. bulloides*, CURL-16069, calibrated Marine13)	
398	46.42	Radiocarbon dating (*G. ruber*, CURL-16070, calibrated Marine13), discarded	
422	46.82	Radiocarbon dating (*G. bulloides*, CURL-16080, calibrated Marine13)	
422	42.92	Radiocarbon dating (*G. ruber*, CURL-16072, calibrated Marine13), discarded	
428	47.79	Radiocarbon dating (*G. bulloides*, CURL-16685, calibrated Marine13)	
428	47.62	Radiocarbon dating (*G. ruber*, CURL-16661, calibrated Marine13)	
476	52.35	Correlation with[47], δD, Vostok ice core	787
518	55	Correlation with[48], globally stacked benthic $\delta^{18}O$	
536	59.59	Correlation with[47], δD, Vostok ice core	877
554	64	Correlation with[48], globally stacked benthic $\delta^{18}O$	
572	67	Correlation with[48], globally stacked benthic $\delta^{18}O$	
608	75	Correlation with[48], globally stacked benthic $\delta^{18}O$	
632	84	Correlation with[48], globally stacked benthic $\delta^{18}O$	
662	94	Correlation with[48], globally stacked benthic $\delta^{18}O$	
692	105	Correlation with[48], globally stacked benthic $\delta^{18}O$	
720	116	Correlation with[48], globally stacked benthic $\delta^{18}O$	
728	123	Correlation with[48], globally stacked benthic $\delta^{18}O$	
736	126	Correlation with[48], globally stacked benthic $\delta^{18}O$	
752	131	Correlation with[48], globally stacked benthic $\delta^{18}O$	
770	135	Correlation with[48], globally stacked benthic $\delta^{18}O$	
800	140	Correlation with[48], globally stacked benthic $\delta^{18}O$	
866	155	Correlation with[48], globally stacked benthic $\delta^{18}O$	

Supplementary Table 3. RAMPFIT[55] output for MD03-2614G *Sporormiella* data.

**Ramp function regression**					
Time interval	1.318-138.286	(39 points)			
xmax	13.230	Xmin	0.520		
mean (x)	7.177	std (x)	4.134		
time spacing maximum		16.338	time spacing minimum		0.435
mean (time spacing)		3.604	std (time spacing)		3.932
Prescribe standard deviation		<48 ka: 3.500	>48 ka: 2.800		
t-search - t1	1.318-138.286	t-search - t2	1.318-138.286		
Fit results	t1 = **43.087**	x1 = **2.041**	t2 = **44.966**	x2 = **9.694**	
**Nonparametric bootstrapping (B = 2000, p = 0.500)**					
	fit	ave	std	med	1.48 mad
t1	43.087	42.951	2.215	43.087	**0.645**
x1	2.041	1.969	0.746	1.996	**0.729**
t2	44.966	45.058	1.268	44.966	**0.851**
x2	9.694	9.715	0.419	9.716	**0.412**

Supplementary Table 4. Percentage values of *Sporormiella* spores and depth and age of each sample analysed from core MD03-2614G, as well as the results from the RAMPFIT routine[55].

Depth (cm)	Age (ka BP)	*Sporormiella* (%)	RAMPFIT output
2	1.317571	0.54	2.040609
10	2.523286	1.45	2.040609
18	3.729	3.23	2.040609
26	5.061444	0.76	2.040609
42	7.439833	3.26	2.040609
80	12.265	0.52	2.040609
155	18.69292	3.01	2.040609
266	30.441	0.89	2.040609
330	35.5492	3.45	2.040609
374	42.652	2.31	2.040609
380	43.087	1.96	2.040609
386	43.522	4.46	3.812241
392	43.957	6.62	5.583888
398	44.392	7.39	7.355519
404	44.96625	12.1	9.694302
410	45.5405	11.34	9.694302
416	46.11475	8.14	9.694302
422	46.689	6.63	9.694302
428	47.297	5.79	9.694302
434	47.92862	8.97	9.694302
446	49.19188	8.28	9.694302
458	50.45512	11.89	9.694302
470	51.71838	12.03	9.694302
482	52.82757	13.23	9.694302
490	53.46433	6.48	9.694302
500	54.26028	9.46	9.694302
512	55.21543	11.56	9.694302
518	55.693	12.38	9.694302
524	56.993	10.76	9.694302
570	66.63133	11.76	9.694302
602	73.7805	4.56	9.694302
620	79.553	10.68	9.694302
635	84.969	10.2	9.694302
650	89.994	5.04	9.694302
680	100.6158	11.7	9.694302
698	107.4104	8.9	9.694302
730	123.7483	4.42	9.694302
752	130.916	12.66	9.694302
790	138.2857	11.11	9.694302
